# Supplementary material for: Folate-Targeted Monodisperse PEG-Based Conjugates Made by Chemo-Enzymatic Methods for Cancer Diagnosis and Treatment
Source: Int J Mol Sci. 2021 Sep 26;22(19):10347. doi: 10.3390/ijms221910347 (PMC8509027; doi:10.3390/ijms221910347)
Supplement: Supplementary file 1 [file ijms-22-10347-s001.zip › ijms-1341260-supplementary.pdf]

Folate-Targeted Monodisperse PEG-Based Conjugates Made by Chemo-Enzymatic Methods for Cancer Diagnosis and Treatment

Krisztina S. Nagy [1], Krisztina Toth [1], Eva Pallinger [2], Angela Takacs [2], Laszlo Kohidai [2], Angela Jedlovszky-Hajdu [1], Domokos Mathe [3,4,5], Noemi Kovacs [4,5], Daniel S. Veres [4], Krisztian Szigeti [4], Kristof Molnar [6], Eniko Krisch [6] and Judit E. Puskas [6,*]

1 Laboratory of Nanochemistry, Department of Biophysics and Radiation Biology, Semmelweis University, Nagyvárad tér 4, 1089 Budapest, Hungary; s.nagykriszti@gmail.com (K.S.N.); toth.krisztina.105@gmail.com (K.T.); hajdu.angela@med.semmelweis-univ.hu (A.J.-H.)
2 Department of Genetics, Cell- and Immunobiology, Semmelweis University, Nagyvárad tér 4, 1089 Budapest, Hungary; pallinger.eva@med.semmelweis-univ.hu (E.P.); angela.takacs1@gmail.com (A.T.); kohlasz2@gmail.com (L.K.)
3 Hungarian Center of Excellence for Molecular Medicine (HCEMM), In Vivo Imaging Advanced Core Facility, Semmelweis University Site, 1094 Budapest, Hungary; mathe.domokos@med.semmelweis-univ.hu
4 Department of Biophysics and Radiation Biology, Semmelweis University, 1094 Budapest, Hungary; kovacsnoi@hotmail.com (N.K.); veres.daniel@med.semmelweis-univ.hu (D.S.V.); szigeti.krisztian@med.semmelweis-univ.hu (K.S.)
5 CROmed Translational Research Centers Ltd., 1094 Budapest, Hungary
6 Department of Food, Agricultural and Biological Engineering, College of Food, Agricultural, and Environmental Sciences, The Ohio State University, 222 FABE, 1680 Madison Avenue, Wooster, OH 44691, USA; molnar.182@osu.edu (K.M.); molnarnekrisch.1@osu.edu (E.K.)
* Correspondence: puskas.19@osu.edu

SUPPLEMENTARY DATA

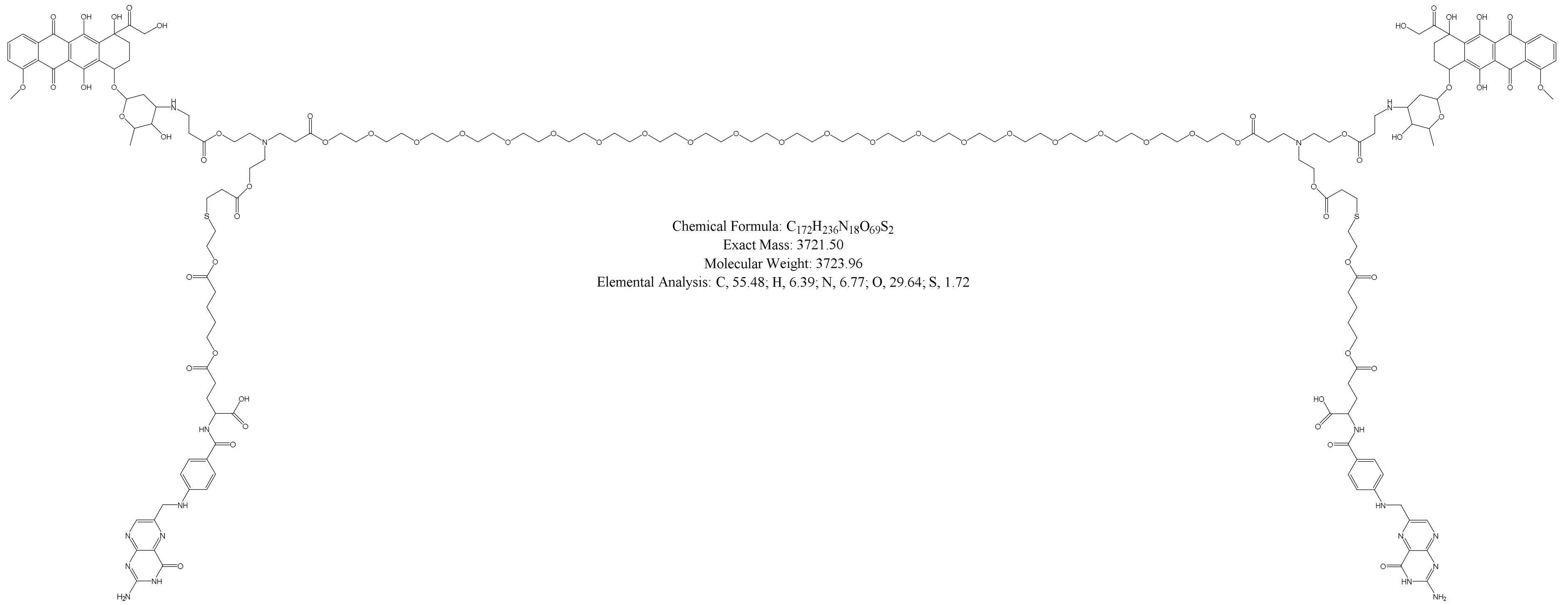

Figure S1. Chemical structure of FA_2-dPEG-DOX_2.

Mice were followed up for 42 days post intra-tumoral single injection. During Days 1, 5, 8, 12, 21, 28, 35 through 42 post injection, three-dimensional MRI scans were obtained to account for exact tumor volumetric changes. We could identify intra-tumoral fluorescence of FA_2-dPEG-DOX_2 at the same excitation/emission wavelengths for 5 days after intra-tumoral administration.

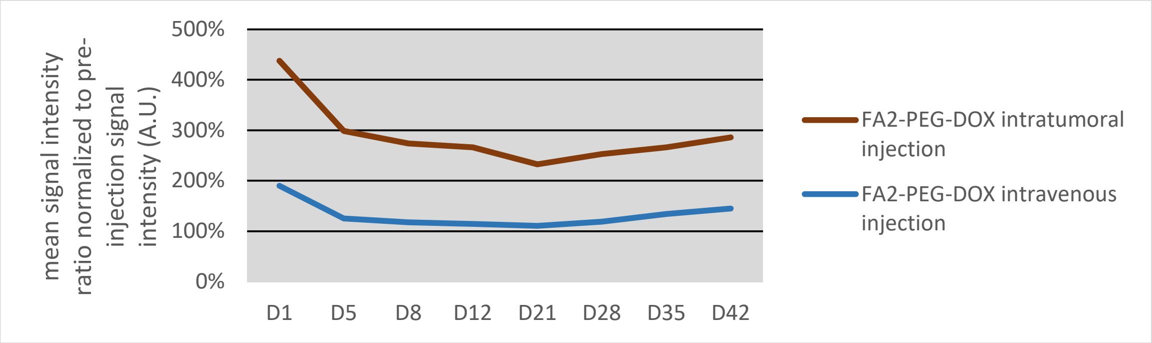

Figure S2. Fluorescent light intensity in percentage to pre-injection light intensity in arbitrary pixel count units (A.U.) over time during 42 days (D) as measured in two-dimensional dorsal fluorescent images of a mouse injected intratumorally/or intravenously.
